# Synchronized High-Speed Vision Sensor Network for Expansion of Field of View †

**DOI:** 10.3390/s18041276

**Published:** 2018-04-21

**Authors:** Akihito Noda, Satoshi Tabata, Masatoshi Ishikawa, Yuji Yamakawa

**Affiliations:** 1 Department of Mechatronics, Nanzan University, 18 Yamazato-cho, Showa-ku, Nagoya 466-8673, Japan; 2 Department of Information Physics and Computing, The University of Tokyo, 7-3-1 Hongo, Bunkyo-ku, Tokyo 113-8656, Japan; Satoshi_Tabata@ipc.i.u-tokyo.ac.jp; 3 Department of Creative Informatics, The University of Tokyo, 7-3-1 Hongo, Bunkyo-ku, Tokyo 113-8656, Japan; Masatoshi_Ishikawa@ipc.i.u-tokyo.ac.jp; 4 Institute of Industrial Science, The University of Tokyo, 4-6-1 Komaba, Meguro-ku, Tokyo 153-8505, Japan; y-ymkw@iis.u-tokyo.ac.jp

**Keywords:** frame synchronization, high-speed vision, networked vision

## Abstract

We propose a 500-frames-per-second high-speed vision (HSV) sensor network that acquires frames at a timing that is precisely synchronized across the network. Multiple vision sensor nodes, individually comprising a camera and a PC, are connected via Ethernet for data transmission and for clock synchronization. A network of synchronized HSV sensors provides a significantly expanded field-of-view compared with that of each individual HSV sensor. In the proposed system, the shutter of each camera is controlled based on the clock of the PC locally provided inside the node, and the shutters are globally synchronized using the Precision Time Protocol (PTP) over the network. A theoretical analysis and experiment results indicate that the shutter trigger skew among the nodes is a few tens of microseconds at most, which is significantly smaller than the frame interval of 1000-fps-class high-speed cameras. Experimental results obtained with the proposed system comprising four nodes demonstrated the ability to capture the propagation of a small displacement along a large-scale structure.

## 1. Introduction

Synchronization of frames is an inherently important issue in stereo vision systems, including binocular and multi-view systems. Three-dimensional (3-D) measurement/reconstruction by stereo vision assumes that multiple frames acquired by multiple cameras are projections of the same 3-D scene. However, if multiple frames of a moving target object were captured by multiple cameras asynchronously, this assumption would be violated because those frames would be projections of the same object but at different positions. For example, suppose that two cameras are capturing images of the same object moving at 100 km/h. A 1 ms error in frame synchronization will correspond to a 28 mm difference in the object positions between the two frames captured by the two cameras. Thus, precise frame synchronization is required for accurate 3-D measurement by stereo vision systems, especially for objects moving at high speed. Without this synchronization, more complicated and costly computations would be required, and greater difficulties would be involved for real-time processing.

Recent high-speed vision (HSV) sensor systems have demonstrated that a higher frame rate is significantly advantageous compared with other lower-frame-rate systems requiring sophisticated algorithms. The advantages include robust and reliable processing, hardware-based implementations with reduced footprint [1], and high-speed computation suitable for real-time feedback control loops [2] and for real-time 3-D reconstruction [3]. Those systems include a single vision sensor or a few tightly coupled vision sensors connected to a single central computer via dedicated interfaces, such as Camera Link.

Another significant approach is event-driven vision sensors [4]. A stereo vision system with an event-matching algorithm has also been reported [5]. Event-driven sensors can generate sparse event data at very high frequency, and the computational cost of matching algorithm can also be reduced compared with conventional frame-based vision sensors. However, this approach requires uncommon special hardware and event matching among networked event-driven vision sensors at large scale will be a significant challenge.

**Figure 1:**
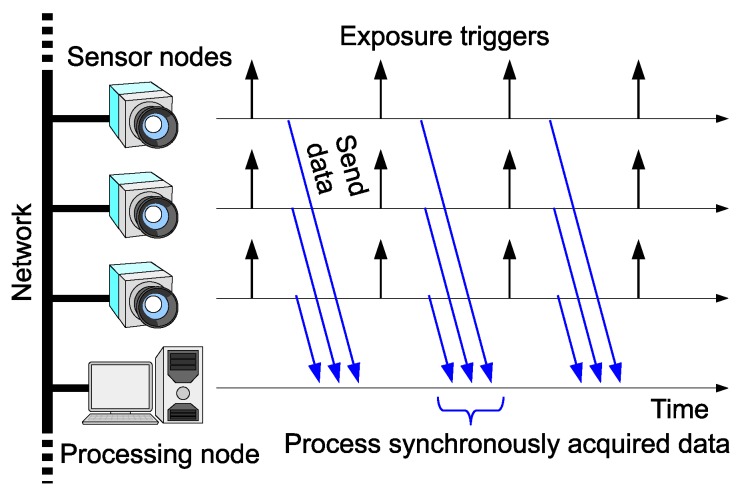
Synchronized HSV sensor network. Frame acquisition is synchronized among sensor nodes across the network.

**Figure 2:**
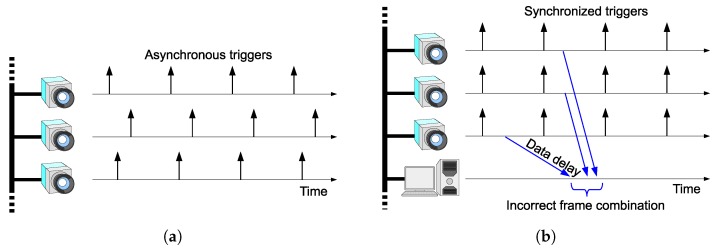
Frame synchronization problem with networked cameras. (**a**) Network latency directly degrades the synchronization accuracy if the trigger signals are transferred via the network. (**b**) Even if the frame acquisition timing is precisely synchronized, the data received via the network can be shuffled in a conventional packet-based network with random delays.

This paper addresses frame synchronization among multiple networked high-speed cameras, as depicted in Figure 1. The motivation of this work is to bring scalability to HSV sensors by connecting the vision sensor nodes via Ethernet. Network latency can cause shutter synchronization errors and incorrect frame combination, as shown in Figure 2a,b respectively. We will present a scheme to avoid these problems.

To avoid these two errors, a clock-synchronization-based scheme can be used for ordinary vision systems operating at 30 frames-per-second (fps) [6]. The synchronization accuracy of the network time protocol (NTP) is millisecond-order, which is not acceptable for HSV sensors with a frame rate of hundreds-of-frames per second or higher (i.e., intervals of less than a few milliseconds). In this work, a real-time frame synchronization scheme for networked HSV sensors is presented, by using Precision Time Protocol (PTP). PTP can achieve sufficiently precise clock synchronization compared with the frame interval of 1000-fps class vision sensors, as explained in Section 5. Note that this is beneficial not only for 1000-fps class systems, but also for 30-fps vision systems that require precise frame synchronization among a large number of cameras networked through Ethernet.

Since Ethernet is highly flexible, the proposed scheme can be applied to a range of systems, from small-scale indoor systems to large-scale multi-room/multi-floor indoor systems where the global positioning system (GPS) cannot be used for synchronization, and to outdoor environments where an illumination-based scheme [7] is not suitable. Such scalability is a significant advantage of the scheme. Notice that the advantage is not limited to physically large systems. Even for a small-scale system, e.g., a desktop-scale system, the scalability and flexibility will be a significant advantage for handling a large number of vision sensor nodes.

The rest of the paper is organized as follows. The motivation and contribution of this work is explained in Section 2. Section 3 describes the frame synchronization algorithm, and an experiment involving a binocular system is presented in Section 4. Section 5 examines the synchronization error through a theoretical analysis. Experiments with a four-node networked vision system are presented in Section 6. Finally, the paper is concluded in Section 7.

## 2. Motivation and Contribution of the Work

Visual-based sensing has the significant advantage that it can remotely/contactlessly monitor objects. Therefore, it can be applied to observation of objects and phenomenon that are fragile and/or sensitive to disturbance, such as microorganisms [8,9]. As another example, in monitoring of civil engineering structures, the visual-based sensing approach can reduce or eliminate the need for contact-based sensing devices [10]. In particular, the HSV-based approach can replace manual inspections and can significantly improve the inspection speed and efficiency [11].

One of the major motivations for using HSV sensors is their ability to track the high-speed motion of objects. However, only a single HSV sensor with a fixed field of view (FOV) will provide insufficient information, because an object moving at high speed can pass through the FOV within a very short period of time.

Assume a series of captured images of an object linearly moving at a speed of 1 pixel-per-frame and with a frame interval of 1 ms. It moves from one side to the other in a 1000-pixel-long FOV within 1 s. To monitor the object for a longer time, the FOV has to move so as to track the object. To move the FOV in this way, an active pan/tilt camera [2] and a combination of galvanometer mirrors [12] have been proposed.

**Figure 3:**
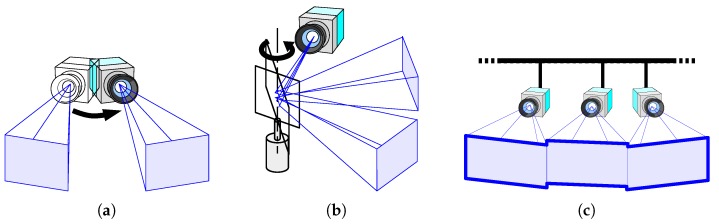
Approaches for expanding the field-of-view: (**a**) Mechanically turning the camera itself, (**b**) changing the gaze direction using a galvanometer mirror, and (**c**) networking multiple cameras.

In this work, we propose another approach, namely, networking multiple fixed HSV sensors. A networked HSV sensor system can provide a FOV significantly larger than that of the individual FOVs of multiple cameras connected via a network, as shown in Figure 3. This is useful not only for observing fast-moving small objects but also for observing moving parts of objects that are larger than each FOV. An example of the kind of application presented here is monitoring the displacement propagation on a one-dimensional (1-D) structure, such as a rod or a string. Another advantage of the networked HSV sensors is the ability to avoid occlusions by using overlapped sections of individual FOVs seen from different camera positions included in the overall FOV [13].

This paper includes a few details that have been reported in our previous conference papers. Our previous reports include a networked HSV sensor system for tracking a miniature robot car [14], a laser spot moving at 30 m/s [14], and a fast-moving table tennis ball [15]. In those previous reports, the system was limited to a binocular system, i.e., two HSV sensors.

The major contributions of this paper are the following two points. The first is to clarify the theoretical aspect of shutter synchronization in a networked HSV sensor system. It helps to decide the parameters used in PTP software to achieve required shutter synchronization precision. The second is to present an application example of a networked HSV sensor system consisting of more than two HSV sensors as a proof-of-concept demonstration. The proposed shutter synchronization scheme can be applied to HSV networks at large scale, thanks to the scalable Ethernet-based clock synchronization protocol, PTP.

## 3. Frame Synchronization

This section examines the problem of frame synchronization.

Figure 2a shows a shutter synchronization error among multiple vision sensor nodes. Asynchronous free-running cameras will cause such an error. For precise stereo processing, the shutter triggers should be synchronized as shown in Figure 1. Another possible approach is to use stroboscope illumination [16], which enables acquisition of synchronized images while the cameras are running asynchronously. This scheme enables frame synchronization; however, correct selection of images for stereo processing in a remote node is not guaranteed in networks with random delays, as shown in Figure 2b. To avoid this kind of misselection, each frame has to be timestamped at each vision sensor node prior to being sent. The remote processing node can choose correct frame combinations based on the timestamps. This scenario requires clock synchronization among the vision sensor nodes with negligible errors significantly smaller than the frame interval. The motivation for this work is to provide a scheme that can realize this scenario. For this purpose, we propose to trigger each camera based on the local clock inside the vision sensor node and to synchronize the clocks across the network.

**Figure 4:**
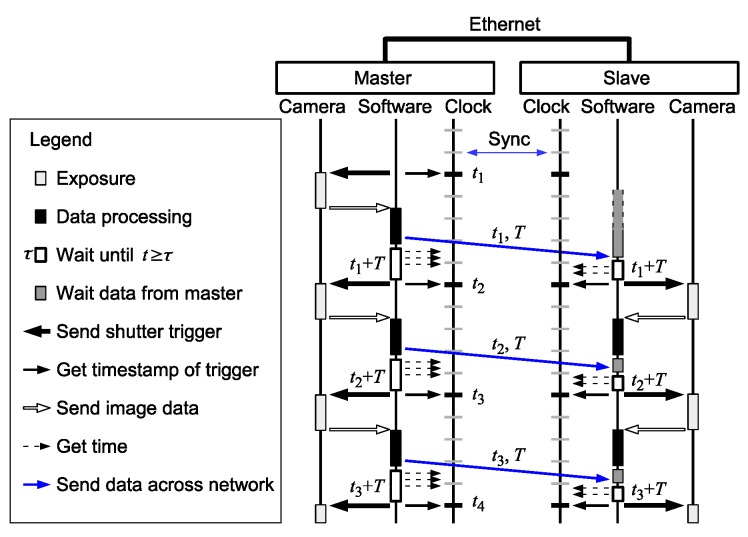
Proposed shutter synchronization scheme. The shutter of each camera is locally controlled based only on the local clock, thus avoiding network delays. The clocks are globally synchronized over Ethernet. The shutter trigger at the slave is determined based on the timestamps ($t_{1},t_{2},\ldots$) and the frame interval (*T*) sent from the master. Although the data from master arrives after a network delay, the trigger timing calculated in the slave does not depend on the delay.

A sequence diagram of the proposed scheme is shown in Figure 4. For simplicity, a single slave is shown. In a multi-slave system, the same process will be performed for each slave. The master node initiates the shutter timing, and the slaves follow it. To prevent the network latency affecting the shutter synchronization precision, the trigger signals are not directly transferred across the network.

The shutter of each camera is locally controlled based only on the local clock and is not affected by network delays. The clocks of the master and the slave are globally synchronized over Ethernet. The clock synchronization error is considerably smaller than the network delays and the frame interval. Due to the small synchronization error, the master and the slave refer to virtually the same clock with negligible delays in the processors.

The shutter trigger at the slave is determined based on the shutter timestamp and frame interval information sent from the master, as described below. When the first shutter is triggered, the master refers to the clock and sends the timestamp of the trigger, $t_{1}$, and the frame interval, *T*, to the slave via Ethernet. The sent data arrives at the slave after a network delay. Both the master and the slave trigger the next shutter at time $t=t_{1}+T$ on their own clock. Thus, provided that the two clocks are synchronized, the shutter triggers are synchronized, and they can acquire image frames with synchronized timestamps. Note that *T* does not necessarily have to be transferred every time. It can also be calculated in the slave by using two adjacent timestamps previously received, e.g., $T=t_{2}-t_{1}$, or can be precoded in every node, because it is typically a fixed value. For dynamically changing frame rate, the data of interval *T* sent from the master have to arrive at the slave prior to the next shutter timing, otherwise the slave’s shutter timing deviates from the master’s.

The proposed scheme can be applied to multi-slave systems without any modification. Multiple slave clocks can be synchronized by PTP. The information about the master shutter timestamps and frame interval can be sent from the master to all the slaves. Based on the synchronized clock and the provided master shutter information, all the slaves can uniformly implement the shutter synchronization scheme.

## 4. Binocular Experimental System

**Figure 5:**
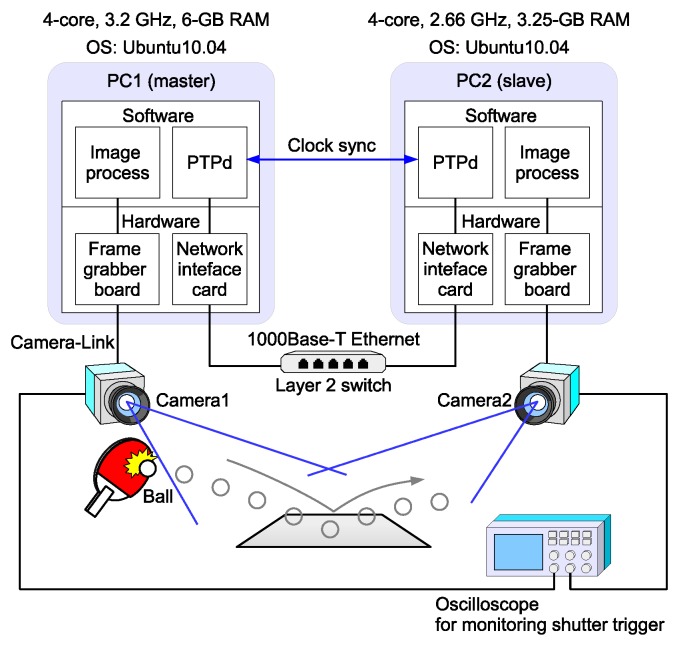
Illustration of experimental system. PC 1 and PC 2 are connected via Ethernet, and the clocks are synchronized by PTP. Cameras 1 and 2 are controlled by PCs 1 and 2, respectively. Shutter control signals fed to the cameras from the PCs are monitored with an oscilloscope.

This section presents the experimental system configuration and measurement results obtained with this system. The details presented here have been reported in part in our conference paper [15]. The experimental system, illustrated in Figure 5, comprises two vision nodes: a master and a slave. Each node consists of a high-frame-rate camera (EoSens MC1362) and a controller PC. The slave PC’s clock is synchronized to the master PC’s with a negligible error of a few microseconds. The PTP daemon (PTPd) for Linux OS [17] is executed as a background process and is independent of the image capturing/processing software.

A frame grabber board (microEnable IV-AD4-CL, Silicon Software GmbH, Mannheim, Germany) is installed in the PC for controlling the camera. The camera resolution was set at 640 × 512 pixels. The camera shutter was triggered by the frame grabber board, and the trigger was controlled by software. The software could get the timestamp immediately after triggering the shutter.

**Figure 6:**
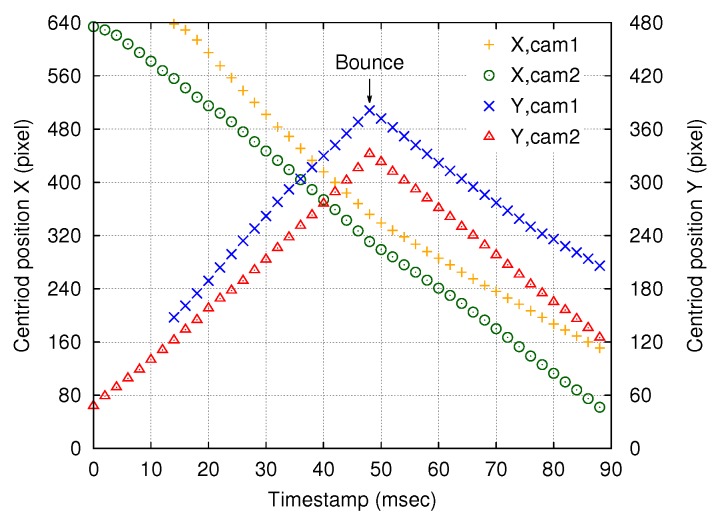
Time-series plot of calculated centroid positions *X* and *Y*, which represent the positions of the moving ball in the 640 × 512-pixel images acquired by cameras 1 and 2 at 500 fps. The timestamp at the first ball detection by camera 2 was used as the time origin, t=0. The timestamp error between the two nodes for each frame was less than 10 μs. A ball bounce was observed at t=48.0 ms in both master and slave images.

**Figure 7:**
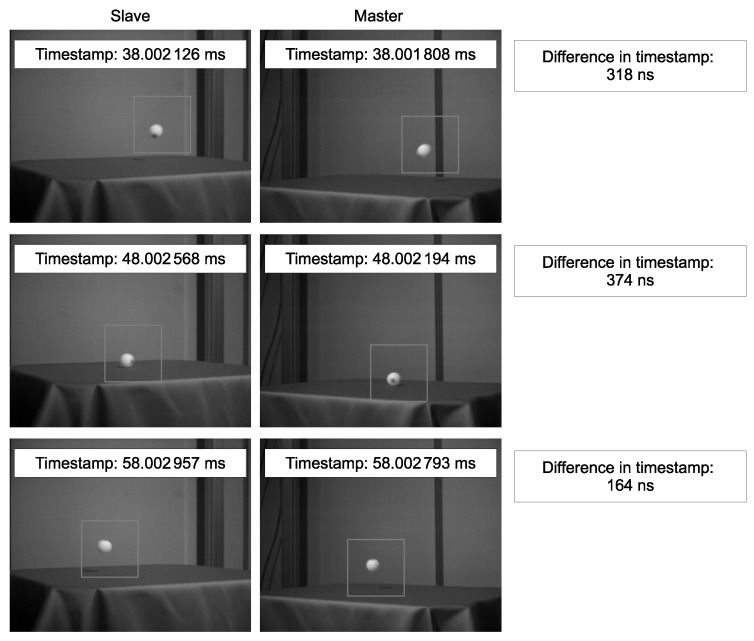
Some of the images acquired by the two vision sensor nodes. The left-hand images were acquired by the slave node, and the right-hand images were acquired by the master node. The images and the corresponding timestamps provided by the PCs are shown. The gray box in each frame represents the region of interest.

The time-series calculated ball positions and examples of actual acquired images are shown in Figure 6 and Figure 7, respectively. The maximum values of $y_{1}$ and $y_{2}$, which represent the bounce of the ball, appeared at the same time, namely, at a timestamp of 48 ms. Thus, precise shutter synchronization across the network was demonstrated.

The tracking operation was performed as follows. First of all, the background image was captured. In the tracking routine, the marker image was extracted by background subtraction. The obtained image was binarized, and the image centroid (X,Y) was calculated as follows:

(1) $$\begin{array}{c} X=m_{1,0}/m_{0,0},\;Y=m_{0,1}/m_{0,0}, \end{array}$$

where $m_{i,j}$ represents the (i,j)-th order image moments expressed as

(2) $$\begin{array}{c} m_{i,j}=\sum_{x}\sum_{y}x^{i}y^{j}I\left(x,y\right). \end{array}$$

HereOnce the marker image was captured and the centroid (X,Y) was successfully calculated, a sub-frame region of interest (ROI) was set around the centroid. The ROI size was set to be 200 × 200 pixels, which is 1/8 as small as the original 640 × 512 pixels, to reduce the computational load.

## 5. Skew

This section presents a theoretical description of skew of the shutter trigger. As described below, the skew does not depend on the network delay. Here, we also consider a single pair of nodes, i.e., a master and a slave.

**Figure 8:**
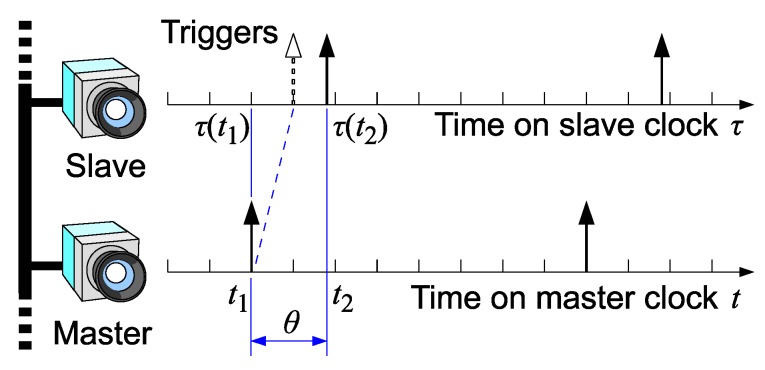
Master’s and slave’s clocks and shutter skew. Because the master trigger timing $t_{1}$ and the slave trigger $\tau (t_{2})$ are respectively measured based on the master and the slave clocks, the actual time difference, $t_{2}-t_{1}$, in the system is unknown.

In the proposed scheme, the shutter synchronization accuracy depends on the clock synchronization accuracy. The clock skew and shutter skew are illustrated in Figure 8.

**Figure 9:**
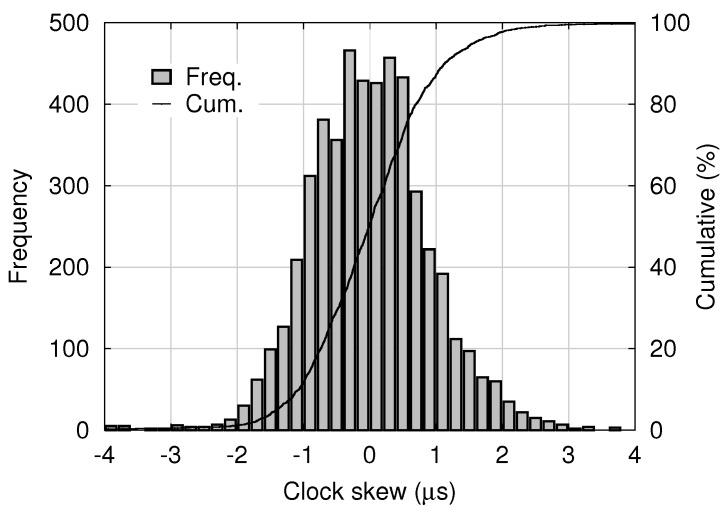
Distribution of clock skew of the slave with respect to the master.

Here we define the clock skew s(t) as follows:

(3) $$\begin{array}{c} s(t)=\tau (t)-t, \end{array}$$

where *t* represents the time measured on the master clock, and τ(t) denotes the time measured on the slave clock at the master time *t*. The skew *s* is estimated and dumped as “offset from master” by the PTPd process running on the slave node. A histogram of estimated skew samples contained in 5000 consecutive log entries dumped by PTPd in the experimental system is shown in Figure 9. The mean and standard deviation were 0.015 μs and 0.98 μs, respectively.

**Figure 10:**
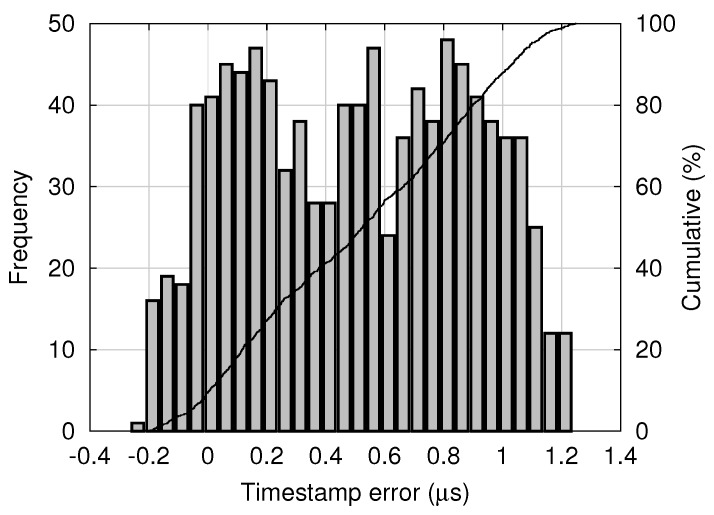
Distribution of timestamp error between the master and the slave.

Let us define a timestamp error ϕ between a pair of “synchronized” frames, i.e., ideally acquired at the same time but actually at slightly different times, as follows:

(4) $$\begin{array}{c} \phi \left(t_{2}\right)=\tau \left(t_{2}\right)-t_{1}, \end{array}$$

where $t_{1}$ and $\tau (t_{2})$ respectively represent the trigger timing of the master and that of the slave. $t_{1}$ is measured on the master clock and $\tau (t_{2})$ is measured on the slave clock. Note that $t_{2}$, the time corresponding to $\tau (t_{2})$ on the master clock, is not directly known. A histogram of timestamp errors obtained in the experimental system is shown in Figure 10.

Let $\theta (t_{2})$ denote the shutter skew, that is, the time difference of the slave shutter with respect to the master shutter, measured on the master clock:

(5) $$\begin{array}{c} \theta \left(t_{2}\right)=t_{2}-t_{1}. \end{array}$$

From Equations (3)–(5), we obtain

(6) $$\begin{array}{c} \theta \left(t_{2}\right)=\tau \left(t_{2}\right)-s\left(t_{2}\right)-t_{1}=\phi \left(t_{2}\right)-s\left(t_{2}\right). \end{array}$$

In the proposed system, although the slave shutter timing $t_{2}$ cannot be directly measured on the master clock, its statistical distribution can be calculated from the probability density functions (PDFs) of ϕ and *s*. Let $f_{\Phi }\left(\phi \right)$ and $f_{S}\left(s\right)$ represent the PDFs, i.e.,

(7) $$\begin{array}{ccc} \mathrm{Pr}[a\leq \phi \leq b]\; & = & \;\int_{a}^{b}f_{\Phi }\left(\phi \right)d\phi \end{array}$$

(8) $$\begin{array}{ccc} \mathrm{Pr}[a\leq s\leq b]\; & = & \;\int_{a}^{b}f_{S}\left(s\right)ds, \end{array}$$

where Pr[·] denotes the probability. Provided that ϕ and *s* are independent, recalling that θ=ϕ−s and using the PDF of negative *s*, $f_{\Sigma }\left(-s\right)=f_{S}\left(s\right)$, the PDF of θ is calculated as the convolution of $f_{\Phi }$ and $f_{\Sigma }$:

(9) $$\begin{array}{ccc} f_{\Theta }\left(\theta \right)\; & = & \;\left(f_{\Phi }*f_{\Sigma }\right)\left(\theta \right)\\ \; & = & \;\int f_{\Phi }\left(\theta -x\right)f_{\Sigma }\left(x\right)dx\\ \; & = & \;\int f_{\Phi }\left(\theta -x\right)f_{S}\left(-x\right)dx. \end{array}$$

For example, assume that $f_{\Phi }$ and $f_{S}$ are normal distributions with means $\mu_{\Phi }$ and $\mu_{S}$ and variances $\sigma_{\Phi }^{2}$ and $\sigma_{S}^{2}$, respectively:

(10) $$\begin{array}{ccc} f_{\Phi }\left(\phi \right)\; & = & \;\frac{1}{\sqrt{2\pi \sigma_{\Phi }^{2}}}\exp\left(-\frac{{\left(\phi -\mu_{\Phi }\right)}^{2}}{2\sigma_{\Phi }^{2}}\right) \end{array}$$

(11) $$\begin{array}{ccc} f_{S}\left(s\right)\; & = & \;\frac{1}{\sqrt{2\pi \sigma_{S}^{2}}}\exp\left(-\frac{{\left(s-\mu_{S}\right)}^{2}}{2\sigma_{S}^{2}}\right). \end{array}$$

In this case, $f_{\Theta }$ is also a normal distribution with mean $\mu_{\Phi }-\mu_{S}$ and variance $\sigma_{\Phi }^{2}+\sigma_{S}^{2}$, as follows:

(12) $$\begin{array}{ccc} f_{\Theta }\left(\theta \right)\; & = & \;\frac{1}{\sqrt{2\pi (\sigma_{\Phi }^{2}+\sigma_{S}^{2})}}\exp\left(-\frac{{\left(\theta -\mu_{\Phi }+\mu_{S}\right)}^{2}}{2(\sigma_{\Phi }^{2}+\sigma_{S}^{2})}\right). \end{array}$$

For this normal distribution, 99.7% of all shutter skews are within the range of $\mu_{\Phi }-\mu_{S}\pm 3\sqrt{\sigma_{\Phi }^{2}+\sigma_{S}^{2}}$. For example, using the means and the standard deviations of the clock skew and the timestamp error shown in Figure 9 and Figure 10, respectively, the range is calculated as 0.62±3.2 μs. Note that the timestamp error shown in Figure 10 seems different from a normal distribution, and it will require only a slight modification. According to Chebyshev’s inequality [18], 99% of random variables with an arbitrary distribution are within a range of μ± 10 σ, where μ and σ denote the mean and the standard deviation, respectively. Thus, in the experimental system, the shutter skew is almost within a range of ±10 μs.

The above theoretical analysis provides information on acceptable clock error distribution. Although the analysis does not directly give the parameters in PTPd including the PID gains and the frequency of message exchange (which also depend on the network configuration), it helps to decide those parameters to achieve required shutter synchronization precision.

**Figure 11:**
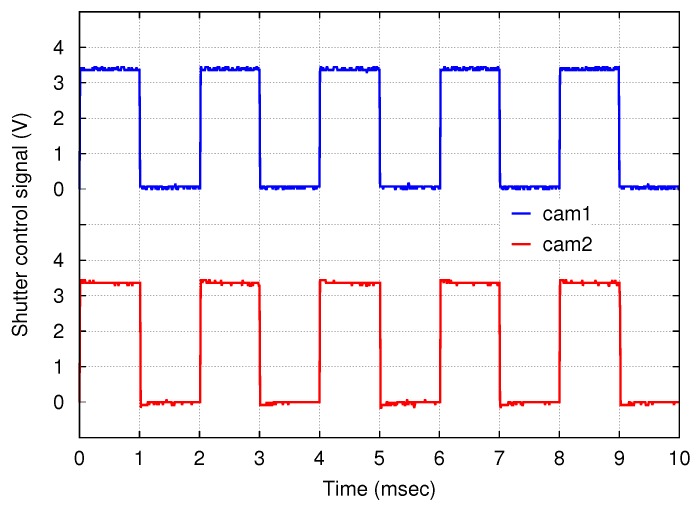
Shutter control signals of cameras 1 and 2, measured by a 2-channel oscilloscope at the same time. The down edges of the signals, which trigger the exposure of the cameras, are synchronized to within negligible errors of less than 10 μs.

The shutter control signals of both cameras were measured with an oscilloscope, and the observed waveforms are shown in Figure 11. Rectangular waves with a frequency of 500 Hz and a duty cycle of 50% were observed. The offset between the down edges of the two signals, i.e., the shutter skew, was less than 10 μs.

Such a small shutter skew will be generally achieved with networked HSV sensor systems based on the proposed scheme. As described in Section 3, the routine for shutter trigger synchronization involves just acquiring the current time and comparing it with the next trigger timing. Therefore, the timestamp error, i.e., the error between the calculated next trigger timing and actual timing when the routine was left, is at most a few microseconds. The clock skew is roughly within 10 μs [19]. Thus, the shutter skew of the proposed scheme is at most a few tens of microseconds. This is an acceptable error for 1000-fps-class HSV sensor networks.

## 6. Applications and Discussions

This section presents example applications of the proposed networked vision system. Using four HSV sensor nodes, displacement propagation on a 1-D structure was observed. As typical examples, in-phase displacement on a rod and solitary wave propagation on a string are presented.

**Figure 12:**
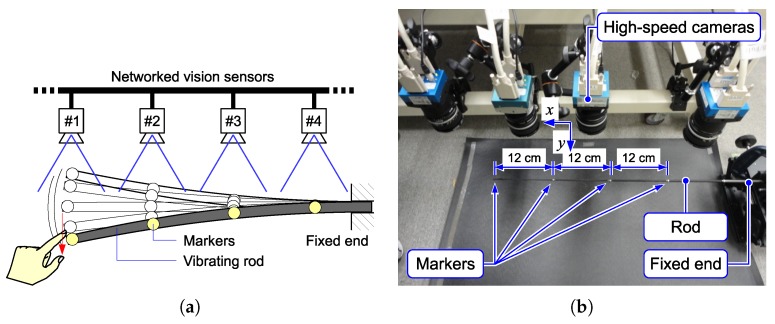
(**a**) Experiment with a vibrating rod. (**b**) Experimental setup.

The first experimental setup is shown in Figure 12. One end of a thin rod (a glass fiber tube) was fixed on a fixture to keep a horizontal attitude, and the other end was free. The tip of the thin rod was flicked by a finger, and its vibration was observed. Four markers were put on the rod, and each marker was captured with an HSV sensor. The HSV sensor detected the marker position based on the procedure described in Section 4.

**Figure 13:**
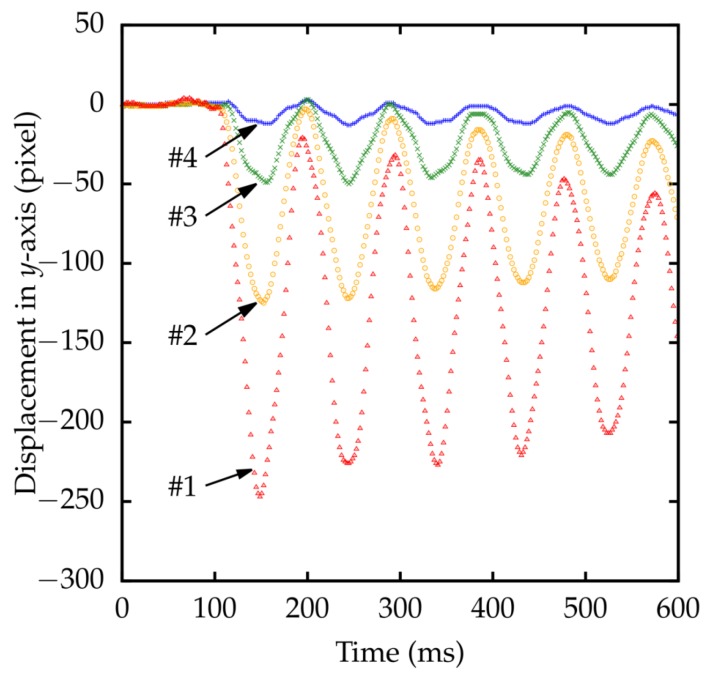
Measured displacement of four markers on a rod. The origin of each marker is its position at time t=0.

Detected marker positions with respect to the *y*-axis of the cameras are shown in Figure 13. Due to the rigidity of the rod, all four markers moved in-phase.

**Figure 14:**
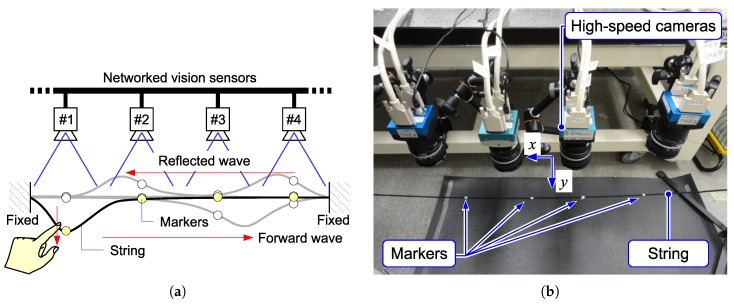
(**a**) Experiment with a vibrating string. (**b**) Experimental setup.

**Figure 15:**
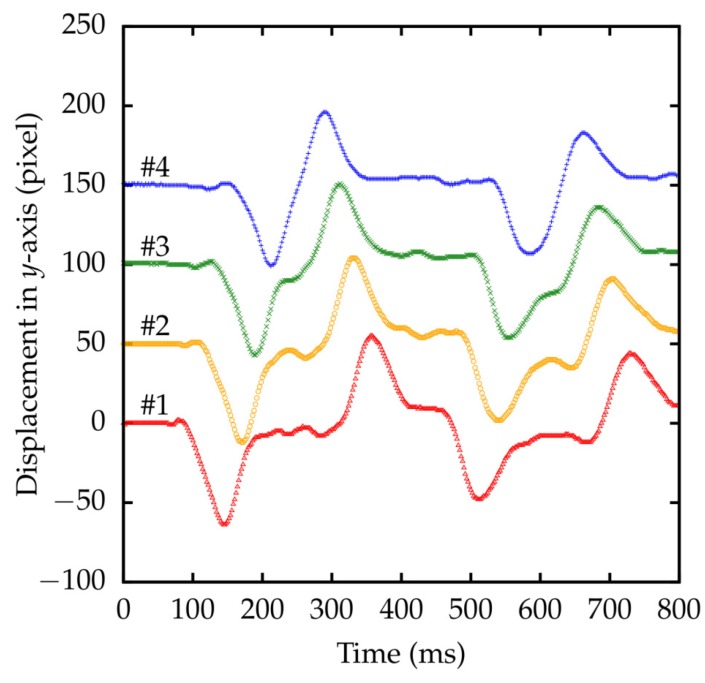
Measured displacement of four markers on a string. The origin of each plot is shifted for visibility.

The second experimental setup is shown in Figure 14. A fabric string was horizontally suspended by fixing both ends on fixtures. Four markers were put on the string. When a section near one end of the string was flicked, the positions of markers were detected by HSV sensors. The result is shown in Figure 15. The displacement was observed as a solitary wave propagation.

The visual-based approach is suitable especially for fragile objects and susceptible phenomenon that should be remotely/contactlessly observed. For example, if the thin rod and the string presented here were loaded with sensor devices, their behavior would be significantly different from their original behavior observed with the proposed system.

**Figure 16:**
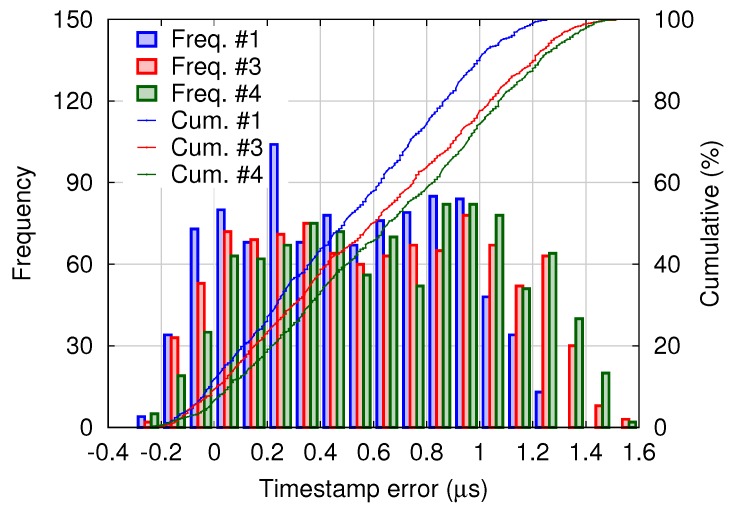
Histogram of the timestamp error between the master (#2) and each slave (#1, #3 and #4).

A histogram of timestamp errors between the master and slaves is shown in Figure 16. For the four-node system, the timestamp error distribution is similar to that of the binocular system shown in Figure 10. Although relatively small-scale experiments were presented here, the proposed scheme can be applied to systems of considerably larger scale.

## 7. Conclusions

In this paper, we have proposed a 500-frames-per-second HSV sensor network that acquires each frame at a timing precisely synchronized across the network. The networked HSV sensors provide an expanded FOV compared with the FOV of each individual camera, without the need for camera turning mechanisms or gaze direction adjusting mechanisms. A theoretical analysis clarified that the shutter skew was at most a few tens of microseconds, which is considerably smaller than the frame interval of 1000-fps-class HSV sensors. Experimental results obtained with the proposed system comprising four nodes demonstrated the ability to capture the propagation of a small displacement along a 1-D structure significantly larger than the FOV of each camera. In-phase oscillatory displacement on a rod and traveling wave of displacement on a string were clearly observed. The precisely shutter-synchronized system provides information of precise phase differences of the oscillatory displacements among different FOVs. PTP, a scalable and precise clock synchronization protocol, supports high scalability of the proposed shutter synchronization scheme. The proposed scheme will enable new applications such as observing and analysing a vibration mode of a large structure across FOVs of a large number of cameras.
